# Uromodulin: A Novel Regulator of the Kidney–Adipose Axis in Diabetic Kidney Disease

**DOI:** 10.3390/ijms27136009

**Published:** 2026-07-04

**Authors:** Linan Cheng, Zheyu Xing, Di Song, Nan Hu, Chunyue Wang, Yuqing Chen

**Affiliations:** 1 Renal Division, Peking University First Hospital, No. 8, Xishiku Street, Xicheng District, Beijing 100034, China; linan1076@163.com (L.C.); zheyu@bjmu.edu.cn (Z.X.); hellosongdi@163.com (D.S.); konan112x@163.com (N.H.); adventofmay@outlook.com (C.W.); 2 Institute of Nephrology, Peking University, Beijing 100034, China; 3 Key Laboratory of Renal Disease, Ministry of Health of China, Beijing 100034, China; 4 Key Laboratory of CKD Prevention and Treatment, Ministry of Education of China, Beijing 100034, China

**Keywords:** uromodulin, lipodystrophy-like phenotype, diabetic kidney disease

## Abstract

The rising burden of diabetic kidney disease (DKD) and its associated lipid abnormalities underscores the need for new mechanistic insights. Uromodulin, a kidney-enriched protein, has been associated with metabolic disorders in human studies, yet its functional role in systemic lipid metabolism remains elusive. In this study, transcriptomic datasets were analyzed to investigate uromodulin expression and biological function in DKD. Subsequently, a diabetic model was induced in *UMOD^+/+^* and *UMOD^−/−^* rats using a combination of a high-fat diet, unilateral nephrectomy, and streptozotocin to assess renal and metabolic phenotypes. Public RNA-seq data indicated that uromodulin expression was downregulated in DKD and was enriched in the fatty acid metabolism pathway. At baseline, *UMOD^−/−^* rats resembled *UMOD^+/+^* rats in terms of growth, routine serum lipids, and major organ function. However, in diabetes, *UMOD^−/−^* rats exhibited higher mortality and pronounced hyperlipidemia. Hyperlipidemia occurred prior to the onset of renal dysfunction. Of note, this exacerbated lipid dysregulation represented a lipodystrophy-like phenotype rather than secondary changes in the pancreas, liver, or circulating cytokines (IL-6, IL-1β, and TNF-α). Moreover, *UMOD^−/−^* rats displayed exacerbated tubular injury and enhanced renal lipid accumulation in DKD relative to *UMOD^+/+^* rats. Collectively, uromodulin protects diabetic rats from death, prevents epididymal white adipose tissue from browning, and attenuates kidney injury. Our findings identify uromodulin as a novel regulator of the kidney–adipose axis.

## 1. Introduction

Diabetes mellitus is a chronic metabolic disorder characterized by persistent hyperglycemia due to defects in insulin secretion or action [1]. Diabetic kidney disease (DKD) is a leading cause of chronic kidney disease (CKD) worldwide, and it is projected to affect nearly 40% of the estimated 640 million individuals with diabetes by 2040 [2]. Despite advances in glycemic control, blood pressure management, and renin–angiotensin system (RAS) blockade, the incidence of DKD continues to rise, highlighting an urgent need to identify novel therapeutic targets [2]. Lipid abnormalities that accompany diabetes are closely linked to the progression of DKD [3]. Early intervention targeting this modifiable risk factor is crucial for delaying the onset and progression of DKD. Recent evidence suggests an association between certain kidney-enriched proteins and systemic metabolic regulation [4], with uromodulin emerging as a candidate of interest [5].

Uromodulin is a kidney-specific protein predominantly synthesized in the thick ascending limb (TAL) and the initial segment of the distal convoluted tubule, and secreted bidirectionally into the tubular lumen and circulation [5]. Uromodulin is traditionally recognized for its local renal functions, including preventing urinary tract infections, inhibiting kidney stone formation, regulating water and electrolyte balance, and immunomodulation [5]. Evidence from an ischemia–reperfusion model in wild-type and *UMOD* knockout (*UMOD^−/^^−^*) mice has shown that uromodulin produced in TAL affects the susceptibility of S3 segments to injury at least in part by regulating MIP-2 expression, suggesting a uromodulin-dependent cross-talk between TAL and proximal tubulars (PTs) [6]. More recently, uromodulin has been found to exert systemic effects outside the kidney, either directly or indirectly through other mediators. Specifically, LaFavers et al. found that uromodulin inhibits oxidative stress in the kidney and systemically by suppressing the activity of transient receptor potential cation channel, subfamily M, member 2 (TRPM2) channel. The rescue experiment using a TRPM2 inhibitor to treat wild-type and *UMOD^−/^^−^* mice with surgery-induced acute kidney injury (AKI) showed that the increased systemic oxidative stress burden was mitigated by treatment with a TRPM2 inhibitor, suggesting that it was at least partially TRPM2-dependent [7]. Micanovic et al. reported that uromodulin regulates granulopoiesis in the bone marrow and the circulation through the proximal tubular epithelial IL-23/IL-17 axis [8]. These findings demonstrate the pleiotropic nature of uromodulin, which exerts diverse effects through a complex network of molecular interactors and signaling pathways, many of which remain to be fully identified.

Observational studies have reported that uromodulin levels are altered in the circulation in diabetic patients [9,10,11]. A prospective study involving 529 patients showed that serum uromodulin was lowest in patients with T2DM at baseline, higher in initially non-diabetic subjects who developed diabetes during follow-up, and highest among non-diabetic patients [11]. Furthermore, several studies have shown associations between uromodulin levels and metabolic disorders [11,12]. Lower serum uromodulin is associated with glucose intolerance in adults [11]. A prospective study of 1088 KORA F4 participants over 6.5 years of follow-up showed that serum uromodulin was independently and inversely associated with metabolic syndrome [12]. Additionally, serum uromodulin was inversely associated with the predominantly pro-inflammatory adipokines chemerin and retinol-binding protein-4, especially in individuals with type 2 diabetes mellitus [13]. These findings suggested that serum uromodulin represents a circulating marker of metabolic syndrome and associated adipose tissue inflammation. However, whether uromodulin can actively participate in systemic lipid metabolism in diabetes remains elusive. Therefore, we employed bioinformatic analyses and a diabetic model in *UMOD^+/+^* and *UMOD^−/−^* rats to investigate the role of uromodulin in systemic lipid metabolism in diabetes.

## 2. Results

### 2.1. Uromodulin Is Downregulated in DKD and Associated with Fatty Acid Metabolism

**Figure 1:**
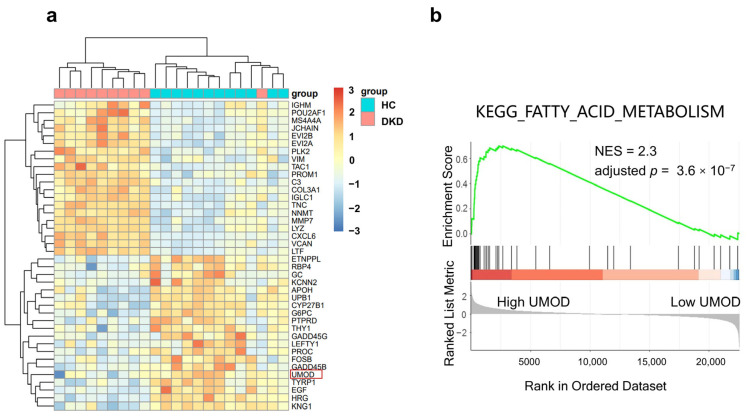
Uromodulin is downregulated in DKD and associated with fatty acid metabolism. (**a**) Analysis of GSE30529 dataset identified *UMOD* as a differentially expressed gene, with downregulation in the DKD group (*n* = 12) compared with HC (*n* = 10), as shown in the heatmap. The red square indicates *UMOD*. (**b**) Gene set enrichment analysis (GSEA) of GSE30529 dataset showed that high *UMOD* expression was enriched in the fatty acid metabolism pathway (NES = 2.3, adjusted *p* = 3.6 × 10^−7^). DKD, diabetic kidney disease; GSEA, Gene set enrichment analysis; HC, health control; NES, normalized enrichment score.

To investigate *UMOD* expression in DKD, we analyzed a tubulointerstitial transcriptomic dataset (GSE30529). Compared with health controls, *UMOD* mRNA levels were significantly reduced in DKD samples (Figure 1a). Gene set enrichment analysis (GSEA) of the GSE30529 dataset showed that high *UMOD* expression was significantly enriched in the fatty acid metabolism pathway (NES = 2.3, adjusted *p* = 3.6 × 10^−7^, Figure 1b), suggesting a unique role for uromodulin in lipid metabolism.

### 2.2. UMOD Deficiency Does Not Affect Baseline Growth, Routine Serum Lipids, or Major Organ Function

**Figure 2:**
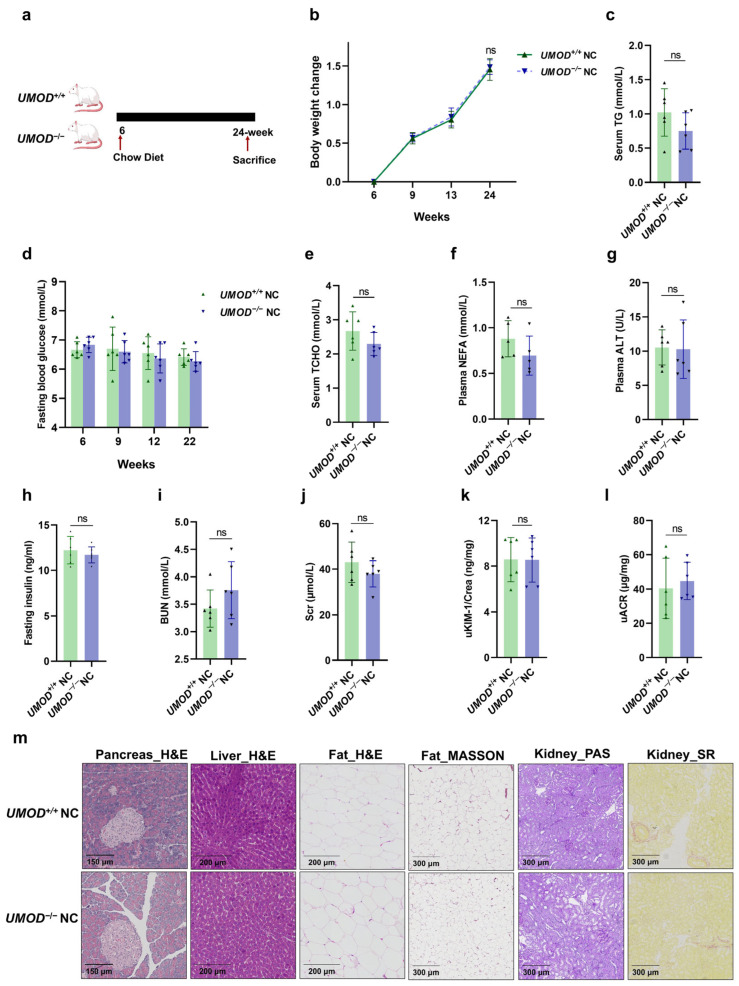
*UMOD* deficiency does not affect baseline growth, routine serum lipids, or major organ function. (**a**) Scheme of the experimental design of rats at baseline. Male *UMOD^+/+^* and *UMOD^−/^^−^* rats (initial age 6 weeks; *n* = 6 per group) were maintained under standard housing and feeding conditions. The arrows indicate the time points of the experimental procedures. The curve of body weight change in *UMOD^+/+^* (*n* = 6) and *UMOD^−/−^* (*n* = 6) naïve control (NC) rats (**b**). Serum triglycerides (TGs, (**c**)), fasting blood glucose (**d**), serum total cholesterol (TCHO, (**e**)), plasma non-esterified fatty acids (NEFAs, (**f**)), plasma alanine aminotransferase (ALT, (**g**)), fasting insulin (**h**), blood urea nitrogen (BUN, (**i**)), serum creatinine (Scr, (**j**)), urinary kidney injury molecule-1-to-creatinine ratio (uKIM-1/Crea, (**k**)), and urinary albumin-to-creatinine ratio (uACR, (**l**)) of *UMOD^+/+^* (*n* = 5–6) and *UMOD^−/−^* (*n* = 5–6) NC rats. (**m**) Images of hematoxylin and eosin (H&E, Bar = 200 μm) staining in the pancreas, liver and epididymal white adipose tissue (eWAT), Masson’s trichrome (Masson, Bar = 300 μm) staining in eWAT, and periodic acid-Schiff (PAS, Bar = 200 μm) staining and Sirius Red staining (Bar = 300 μm) in kidney from *UMOD^+/+^* (*n* = 5) and *UMOD^−/−^* (*n* = 5) NC rats. Data shown represent mean ± SD and were analyzed by unpaired, two-sided *t*-test with Welch correction (**c**–**k**). ns, not significant. ALT, alanine aminotransferase; BUN, blood urea nitrogen; H&E, hematoxylin and eosin; Masson, Masson’s trichrome; NC, naïve control; NEFAs, non-esterified fatty acids; PAS, periodic acid-Schiff; Scr, serum creatinine; STZ, streptozotocin; SR, Sirius Red; TCHO, total cholesterol; TGs, triglycerides; uKIM-1/Crea, urinary kidney injury molecule-1-to-creatinine ratio; UNx, unilateral nephrectomy; uACR, urinary albumin-to-creatinine ratio.

To assess whether *UMOD* deficiency causes baseline abnormalities, we compared *UMOD^−/^^−^* and *UMOD^+/+^* rats under steady-state conditions (Figure 2a). Both genotypes showed comparable body weight growth (Figure 2b) and 100% survival, with no significant differences in fasting blood glucose (Figure 2d), fasting insulin (Figure 2h), plasma alanine aminotransferase (ALT, Figure 2g), or serum lipid parameters, including triglycerides (TGs, Figure 2c), non-esterified fatty acids (NEFAs, Figure 2f), and total cholesterol (TCHO, Figure 2e). Histological examination revealed normal architecture in liver, pancreas, and epididymal white adipose tissue (eWAT) (Figure 2m). Renal function parameters—including serum creatinine (Scr, Figure 2j), blood urea nitrogen (BUN, Figure 2i), urine albumin-to-creatinine ratio (uACR, Figure 2l), and urinary kidney injury molecule-1 (KIM-1, Figure 2k)-to-creatinine ratio —did not differ between genotypes. Periodic Acid-Schiff (PAS) and Sirius Red staining confirmed comparable renal morphology and minimal collagen deposition (Figure 2m). Collectively, *UMOD* deficiency does not affect growth, survival, or the structural and functional integrity of major metabolic organs under baseline conditions up to 24 weeks of age.

### 2.3. Uromodulin Expression Is Altered in Serum, Urine, and the Kidneys of Diabetic UMOD^+/+^ Rats

**Figure 3:**
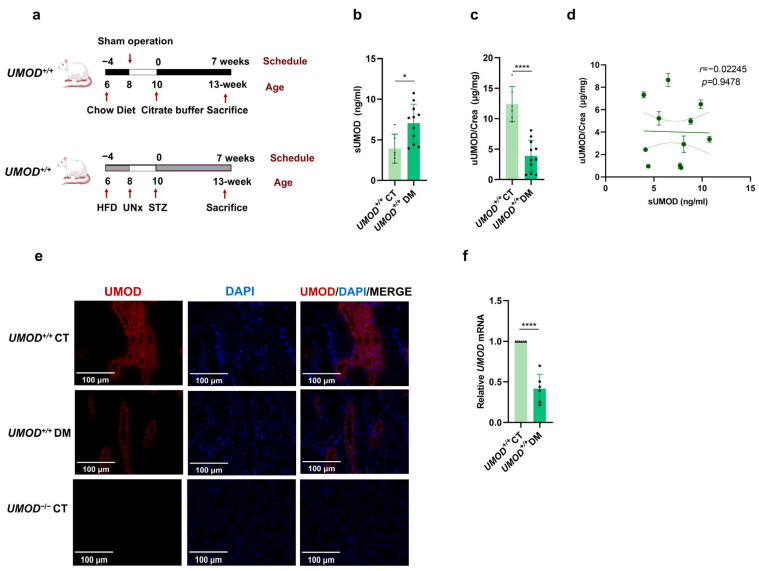
Uromodulin expression is altered in serum, urine, and the kidneys of diabetic *UMOD^+/+^* rats. (**a**) Scheme of the experimental design. Twenty male *UMOD^+/+^* (6 weeks old) were assigned to a blank control (CT, *n* = 6) or diabetes mellitus (DM) group (*n* = 14). Rats in the DM group were fed a high-fat diet (HFD) for 2 weeks, followed by unilateral nephrectomy (UNx). Two weeks after surgery, they received an intraperitoneal injection of 35 mg/kg streptozotocin (STZ) (designated as day 0). HFD feeding was maintained throughout the experimental period. Blank control received a standard diet, sham surgery, and vehicle injection. All rats were sacrificed at week 7 after STZ injection. The arrows indicate the time points of the experimental procedures. Serum uromodulin (sUMOD, (**b**)) and urinary uromodulin-to-creatinine ratio (uUMOD/Crea, (**c**)) of *UMOD^+/+^* CT (*n* = 5) and *UMOD^+/+^* DM (*n* = 11) rats. (**d**) Scatter plot showing the correlation between sUMOD and uUMOD/Crea in diabetic *UMOD^+/+^* rats (*n* = 11). Pearson correlation analysis revealed a non-significant negative correlation between sUMOD and uUMOD/Crea (*r* = −0.02245, *p* = 0.9478). Each dot represents one individual. (**e**) Images of immunolabeling of uromodulin in kidney sections from *UMOD^+/+^* CT (*n* = 3) and *UMOD^+/+^* DM (*n* = 5) rats. (**f**) Quantification of *UMOD* expression in kidney of *UMOD^+/+^* DM (*n* = 5) rats relative to *UMOD^+/+^* CT rats. Data shown represent mean ± SD and were analyzed by unpaired, two-sided *t*-test with Welch correction. * *p* < 0.05 and **** *p* < 0.0001. CT, control; DAPI, 4′,6-diamidino-2-phenylindole; DM, diabetes mellitus; sUMOD, serum uromodulin; uUMOD/Crea, urinary uromodulin-to-creatinine ratio; UMOD, uromodulin.

We next examined uromodulin expression in diabetic *UMOD^+/+^* rats (Figure 3a). Serum uromodulin levels were significantly elevated in diabetic rats compared with non-diabetic controls (Figure 3b). In contrast, urinary uromodulin levels, normalized to creatinine, were markedly reduced (Figure 3c). No significant correlation was observed between serum and urinary uromodulin levels (Figure 3d). Renal *UMOD* mRNA expression was also significantly downregulated in diabetic rats (Figure 3f). Immunofluorescence staining confirmed markedly diminished uromodulin protein expression in the kidney of diabetic *UMOD^+/+^* rats (Figure 3e).

### 2.4. UMOD Deficiency Alters the Systemic Lipid Profile Prior to Renal Dysfunction in Diabetes

**Figure 4:**
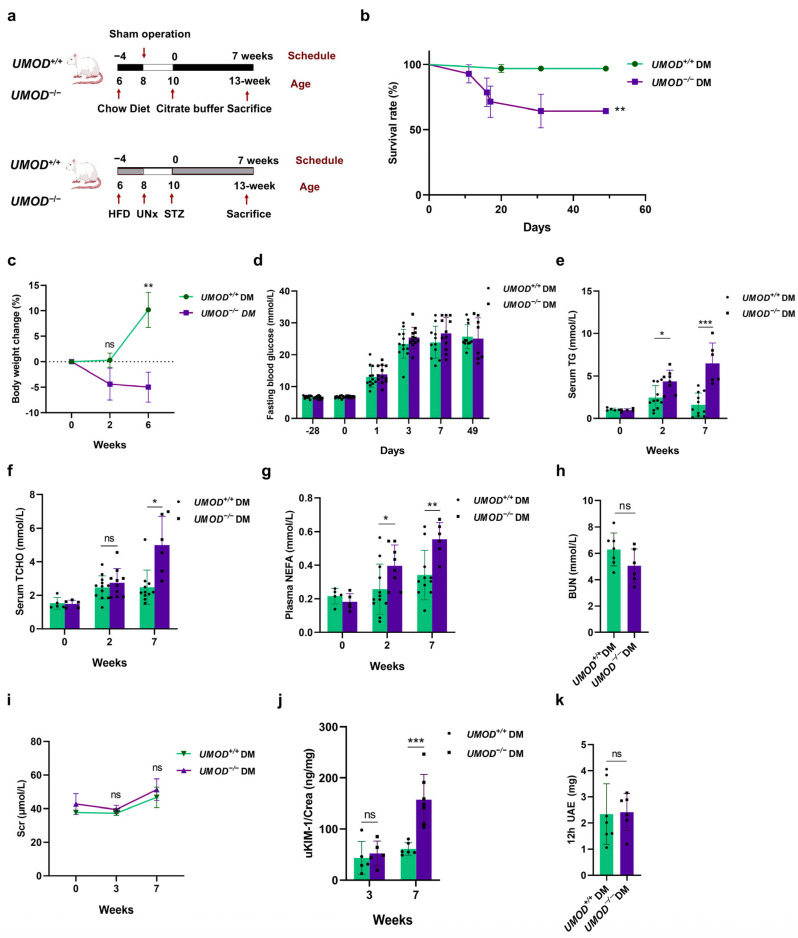
*UMOD* alters the systemic lipid profile prior to renal dysfunction in diabetes. (**a**) Scheme of the experimental design of this study. Twenty male *UMOD^+/+^* and twenty male *UMOD^−/−^* SD rats (6 weeks old) were assigned to a blank control (*n* = 6 per genotype) or diabetes mellitus (DM) group (*n* = 14 per genotype). Rats in the DM group were fed a high-fat diet (HFD) for 2 weeks, followed by unilateral nephrectomy (UNx). Two weeks after surgery, they received an intraperitoneal injection of 35 mg/kg streptozotocin (STZ) (designated as day 0). HFD feeding was maintained throughout the experimental period. Blank control received a standard diet, sham surgery, and vehicle injection. All rats were sacrificed at week 7 after STZ injection. The arrows indicate the time points of the experimental procedures. The curve of survival rate (**b**) and body weight change (**c**) of *UMOD^+/+^* (*n* = 12) and *UMOD^−/−^* (*n* = 13) DM rats. Fasting blood glucose (**d**), serum triglycerides (TGs, (**e**)), serum total cholesterol (TCHO, (**f**)), plasma non-esterified fatty acids (NEFAs, (**g**)), blood urea nitrogen (BUN, (**h**)), serum creatinine (Scr, (**i**)), urinary kidney injury molecule-1-to-creatinine ratio (uKIM-1/Crea, (**j**)), and 12 h urinary albumin excretion (12 h UAE, (**k**)) of *UMOD^+/+^* (*n* = 5–12) and *UMOD^−/−^* (*n* = 5–13) DM rats. The number of animals included in the analyses is depicted in the graphs. Data shown represent mean ± SD and were analyzed by the log-rank test (**b**) or unpaired, two-sided *t*-test with Welch correction (**c**–**k**). * *p* < 0.05, ** *p* < 0.01, *** *p* < 0.001, and ns, not significant. BUN, blood urea nitrogen; DM, diabetes mellitus; HFD, high-fat diet; NEFAs, non-esterified fatty acids; Scr, serum creatinine; STZ, streptozotocin; TCHO, total cholesterol; TGs, triglycerides; uKIM-1/Crea, urinary kidney injury molecule-1-to-creatinine ratio; UNx, unilateral nephrectomy; 12 h UAE, 12 h urinary albumin excretion.

Figure 4a illustrates the experimental workflow. Diabetes was successfully induced in 13 of 14 (92.9%) *UMOD^−/^^−^* rats, compared with 12 of 14 rats (85.7%) in the *UMOD^+/+^* group. Survival rates were significantly lower in diabetic *UMOD^−/^^−^* rats than in *UMOD^+/+^* rats (Figure 4b). Body weight recovered in diabetic *UMOD^+/+^* rats from week 2 post-STZ onward, whereas diabetic *UMOD^−/^^−^* rats exhibited sustained weight loss, with a significant difference in weight gain by week 6 (Figure 4c). Both groups maintained fasting blood glucose levels > 11.1 mmol/L from day 3 post-STZ until the end of the experiment, with no intergroup difference (Figure 4d). Notably, diabetic *UMOD^−/^^−^* rats developed significant dyslipidemia as early as week 2, with elevated TGs (Figure 4e) and NEFAs (Figure 4g). TCHO became significantly elevated by week 7 (Figure 4f). No significant differences were observed in Scr (Figure 4i), BUN (Figure 4h), or 12 h urinary albumin excretion (12h UAE, Figure 4k) between diabetic *UMOD^+/+^* and *UMOD^−/^^−^* groups until week 7. However, urinary KIM-1-to-creatinine ratio, while comparable between groups at week 3, became significantly elevated in diabetic *UMOD^−/^^−^* group by week 7 (Figure 4j).

### 2.5. UMOD Deficiency Does Not Affect the Function of Pancreas, Liver, or Key Inflammatory Markers in Diabetes

**Figure 5:**
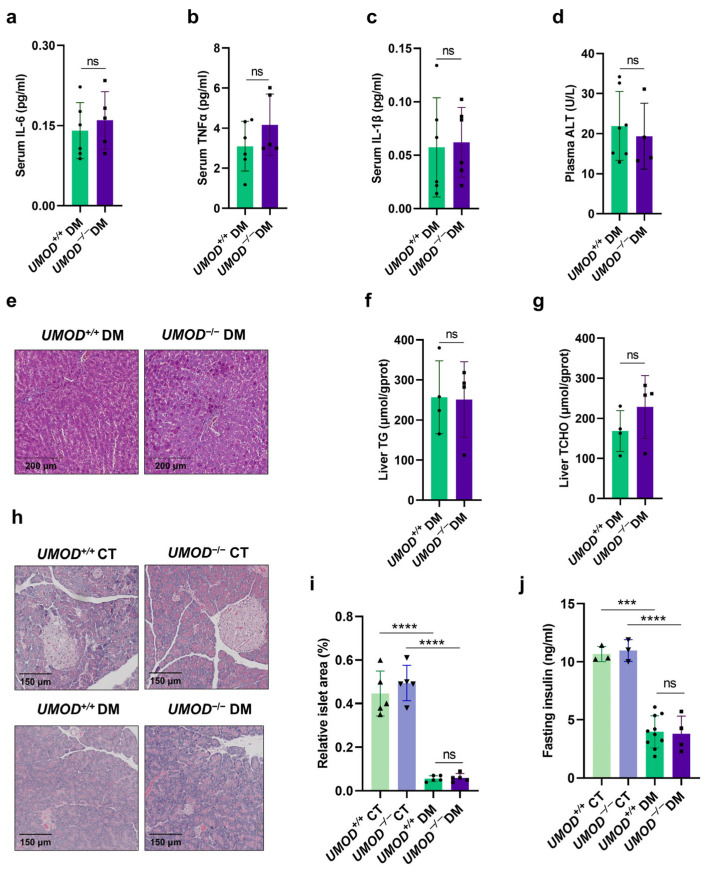
*UMOD* deficiency does not affect the function of pancreas, liver, or key inflammatory markers in diabetes. Serum interleukin-6 (IL-6, (**a**)), serum tumor necrosis factor-alpha (TNF-α, (**b**)), serum interleukin-1 beta (IL-1β, (**c**)), and plasma alanine aminotransferase (ALT, (**d**)) of *UMOD^+/+^* (*n* = 6) and *UMOD^−/−^* diabetes mellitus (DM, *n* = 6) rats. (**e**) Images of hematoxylin and eosin (H&E, Bar = 200 μm) staining in liver from *UMOD^+/+^* (*n* = 5) and *UMOD^−/−^* (*n* = 5) DM rats. Quantification of liver triglyceride (TGs, (**f**)) and total cholesterol (TCHO, (**g**)) levels normalized to total protein in *UMOD^+/+^* (*n* = 4) and *UMOD^−/−^* (*n* = 4) DM rats. (**h**,**i**) Images and quantification of H&E (Bar = 200 μm) staining in the pancreas from *UMOD^+/+^* control (CT, *n* = 5), *UMOD^−/−^* CT (*n* = 5), *UMOD^+/+^* DM (*n* = 5), and *UMOD^−/−^* DM (*n* = 5) rats. (**j**) Fasting insulin of *UMOD^+/+^* CT (*n* = 3), *UMOD^−/−^* CT (*n* = 3), *UMOD^+/+^* DM (*n* = 10), and *UMOD^−/−^* (*n* = 4) DM rats. Data are shown as mean ± SD or median with IQR as appropriate, and were analyzed using an unpaired, two-sided *t*-test with Welch’s correction (**a**,**b**,**f**,**g**), a Mann–Whitney U test (**c**,**d**), or a one-way ANOVA with Tukey’s multiple comparisons test (**i**,**j**). *** *p* < 0.001, **** *p* < 0.0001, and ns, not significant. ALT, alanine aminotransferase; CT, control; DM, diabetes mellitus; IL-1β, interleukin-1 beta; IL-6, interleukin-6; TCHO, total cholesterol; TGs, triglyceride; TNF-α, tumor necrosis factor-alpha.

To explore the mechanism underlying hyperlipidemia in diabetic *UMOD^−/^^−^* rats, we assessed circulating inflammatory markers and key metabolic organs. No significant differences in serum interleukin-6 (IL-6), interleukin-1 beta (IL-1β), tumor necrosis factor-alpha (TNF-α), or plasma ALT were observed between diabetic *UMOD^−/−^* and *UMOD^+/+^* rats (Figure 5a–d). Fasting insulin levels declined in both DM groups compared with controls, with no intergenotype difference (Figure 5j). Liver histology (Figure 5e) and hepatic TG and TCHO content (Figure 5f,g) were comparable between the two diabetic groups. Pancreatic hematoxylin and eosin (H&E) staining showed equivalent STZ-induced islet atrophy (Figure 5h,i). These data indicate that the metabolic phenotype of *UMOD^−/^^−^* rats is not attributable to intergroup differences in pancreatic or hepatic injury or to overt inflammation.

### 2.6. UMOD Deficiency Induces a Lipodystrophy-like Phenotype in Diabetes

**Figure 6:**
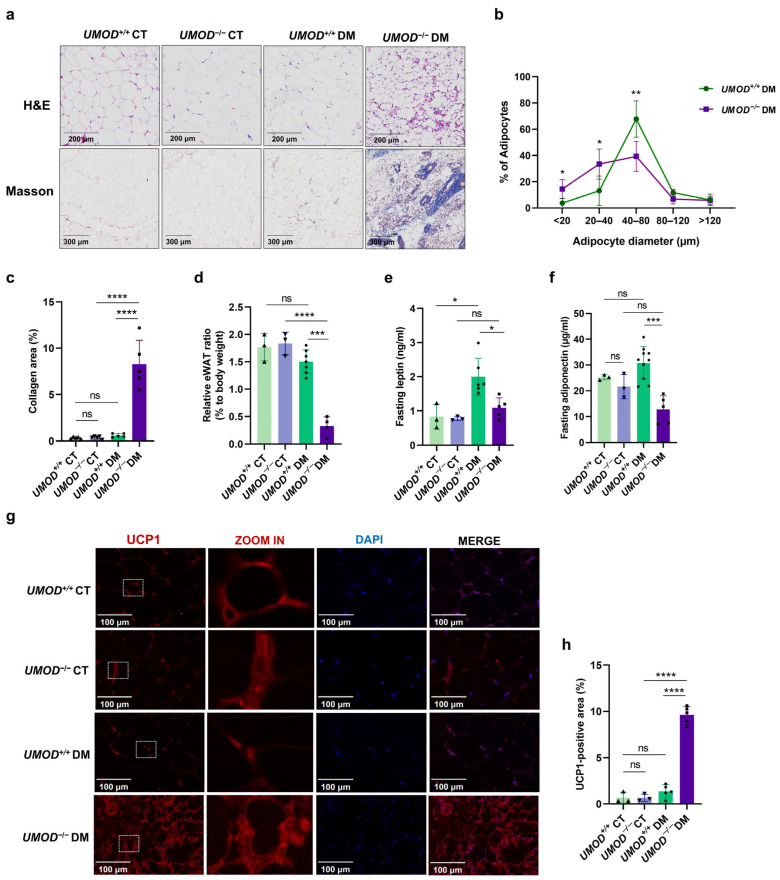
*UMOD* deficiency induces a lipodystrophy-like phenotype in diabetes. Images and quantification of hematoxylin and eosin (H&E, Bar = 200 μm) staining (**a**,**b**) and Masson’s trichrome (Masson, Bar = 300 μm) staining (**a**,**c**) in eWAT from *UMOD^+/+^* control (CT, *n* = 3), *UMOD^−/−^* CT (*n* = 3), *UMOD^+/+^* diabetes mellitus (DM, *n* = 5), and *UMOD^−/−^* DM (*n* = 5) rats. Relative epididymal white adipose tissue (eWAT) ratio (**d**), fasting leptin (**e**), and fasting adiponectin (**f**) in *UMOD^+/+^* CT (*n* = 3), *UMOD^−/−^* CT (*n* = 3), *UMOD^+/+^* DM (*n* = 6–10), *UMOD^−/−^* DM (*n* = 4–5). (**g**,**h**) Images and quantification of immunolabeling of uncoupling protein 1 (UCP1, Bar = 100 μm) in eWAT from *UMOD^+/+^* CT (*n* = 3), *UMOD^−/−^* CT (*n* = 3), *UMOD^+/+^* DM (*n* = 5), and *UMOD^−/−^* DM rats (*n* = 5). Data shown represent mean ± SD and were analyzed by unpaired, two-sided *t*-test with Welch correction (**b**) or one-way ANOVA with Tukey’s multiple comparisons test (**c**–**f**,**h**). * *p* < 0.05, ** *p* < 0.01, *** *p* < 0.001, **** *p* < 0.0001, and ns, not significant. CT, control; DAPI, 4′,6-diamidino-2-phenylindole; DM, diabetes mellitus; eWAT, epididymal white adipose tissue; H&E, hematoxylin and eosin; Masson, Masson’s trichrome; UCP1, uncoupling protein 1.

Diabetic *UMOD^−/^^−^* rats exhibited a marked reduction in eWAT weight compared with diabetic *UMOD^+/+^* rats, whereas eWAT weight in diabetic *UMOD^+/+^* rats did not differ from non-diabetic controls (Figure 6d). This was accompanied by decreased circulating leptin and adiponectin levels (Figure 6e,f). Compared with diabetic *UMOD^+/+^* rats, eWAT from diabetic *UMOD^−/^^−^* rats showed reduced adipocyte size on H&E staining (Figure 6a,b) and more severe fibrosis on Masson’s trichrome staining (Figure 6a,c). Expression of the gene encoding the thermogenic regulator uncoupling protein 1 (UCP1) was elevated in eWAT from diabetic *UMOD^−/^^−^* rats (Figure 6g,h). Collectively, these data demonstrate that *UMOD* deficiency induces a lipodystrophy-like phenotype in diabetes.

### 2.7. UMOD Deficiency Leads to Severe Renal Injury and Lipid Accumulation in Diabetes

**Figure 7:**
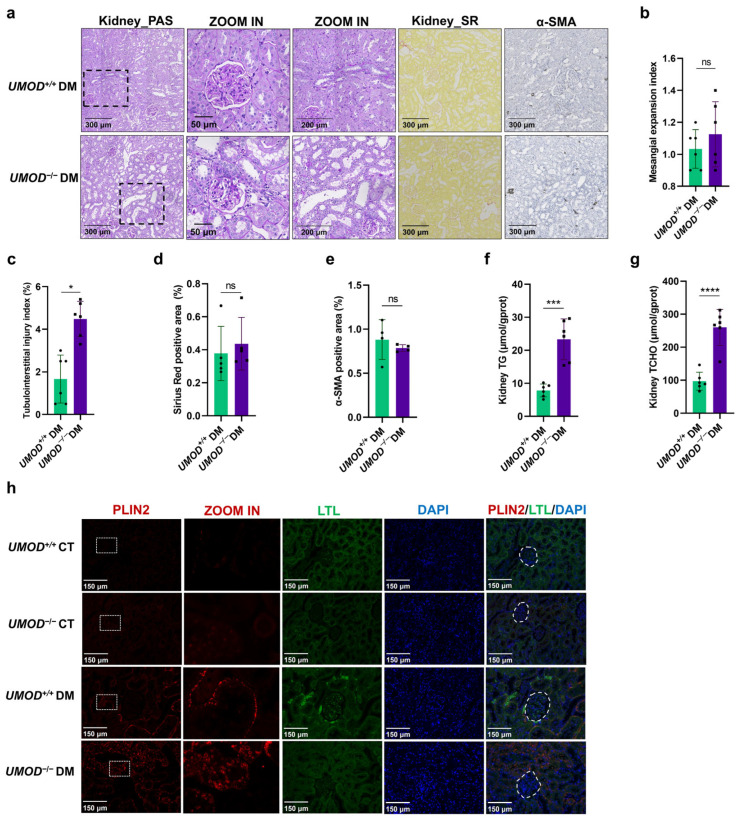
*UMOD* deficiency leads to severe renal injury and lipid accumulation in diabetes. (**a**) Images and quantification of periodic acid-Schiff (PAS, Bar = 200 μm, (**a**–**c**)) staining, Sirius Red staining (Bar = 300 μm, (**a**,**d**)), and alpha-smooth muscle actin (α-SMA, Bar = 300 μm, (**a**,**e**)) immunolabeling in kidney from *UMOD^+/+^* (*n* = 6) and *UMOD^−/−^* (*n* = 4–6) rats with diabetes mellitus (DM). Quantification of renal triglyceride (TGs, (**f**)) and total cholesterol (TCHO, (**g**)) levels normalized to total protein in *UMOD^+/+^* (*n* = 6) and *UMOD^−/−^* DM (*n* = 5–6) rats. (**h**) Images of immunolabeling of perilipin 2 (PLIN2, Bar = 150 μm) in kidney from *UMOD^+/+^* control (CT, *n* = 3), *UMOD^−/−^* CT (*n* = 3), *UMOD^+/+^* DM (*n* = 6), and *UMOD^−/−^* DM (*n* = 5) rats. White circles denote the glomeruli. Data shown represent mean ± SD and were analyzed by unpaired, two-sided *t*-test with Welch correction. * *p* < 0.05, *** *p* < 0.001, **** *p* < 0.01, and ns, not significant. CT, control; DAPI, 4′,6-diamidino-2-phenylindole; DM, diabetes mellitus; LTL, lotus tetragonolobus lectin; PAS, periodic acid-Schiff; PLIN2, perilipin 2; SR, Sirius Red; α-SMA, alpha-smooth muscle actin.

Diabetic *UMOD^−/^^−^* rats developed more severe acute tubular injury than diabetic *UMOD^+/+^* rats, as assessed by PAS staining, while the mesangial expansion index was comparable between groups (Figure 7a–c). Sirius Red staining (Figure 7a,d) and α-SMA immunostaining (Figure 7a,e) showed similar collagen deposition, indicating no difference in interstitial fibrosis at this stage. Renal lipid content was significantly increased in diabetic *UMOD^−/−^* rats, including both TGs (Figure 7f) and TCHO (Figure 7g), accompanied by upregulation of perilipin 2 (PLIN2) expression (Figure 7h). Notably, PLIN2 upregulation was observed predominantly in tubules, with no apparent increase in glomeruli. These findings indicate that *UMOD* deficiency exacerbates tubular injury and promotes renal lipid accumulation without affecting glomerular injury or interstitial fibrosis at this stage.

## 3. Discussion

To our knowledge, this is the first study to reveal an unexpected role of uromodulin as a regulator of adipose tissue homeostasis. Uromodulin is well recognized for maintaining renal homeostasis, yet its role in systemic homeostasis remains unclear [5]. Recent evidence linking uromodulin to metabolic syndrome prompted us to investigate its role in lipid metabolism [12]. Here, we report a multi-hit DKD rat model that meets the Animal Models of Diabetic Complications Consortium (AMDCC) criteria for early-stage DKD rather than advanced DKD, as shown by preserved Scr and BUN, mild mesangial expansion and tubulointerstitial fibrosis, and absence of elevated TNF-α, IL-6, and IL-1β [14,15]. In this model, *UMOD* deficiency causes early-stage hyperlipidemia prior to kidney dysfunction and weight loss, suggesting that hyperlipidemia is an upstream event of these pathological changes. In further analysis of hyperlipidemia and potential underlying causes, our data show that *UMOD* deficiency induces a lipodystrophy-like phenotype, and the exacerbated lipid dysregulation in *UMOD^−/^^−^* rats is not attributable to secondary alterations in the pancreas, liver, or circulating IL-6, IL-1β, and TNF-α. Overall, diabetic *UMOD^−/^^−^* rats present a systemic phenotype involving hyperlipidemia, weight loss, adipose atrophy, mild kidney injury, and increased mortality. The novelty of our study lies in the finding that *UMOD* deficiency is associated with adipose atrophy and increased mortality that are disproportionately severe relative to the mild kidney injury.

**Figure 8:**
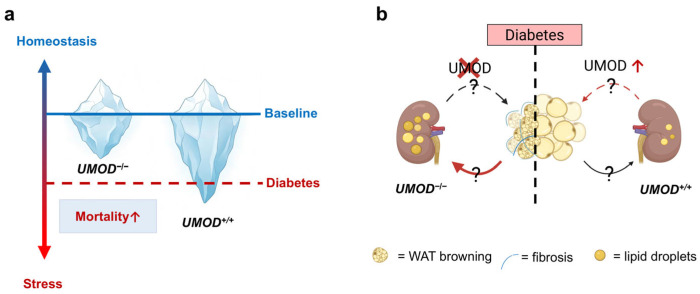
Schematic illustrations of the protective role of uromodulin. (**a**) Iceberg model. *UMOD* deficiency is silent at baseline (similar tips) but predisposes rats to a more severe response to diabetes (exposed by falling water level), resulting in increased mortality. (**b**) Role of uromodulin in adipose homeostasis and kidney protection in diabetic kidney disease (DKD). Under diabetic conditions, uromodulin prevents browning and fibrosis of epididymal white adipose tissue (eWAT) and attenuates renal lipid accumulation. Solid arrows indicate direct effects. Dashed arrows indicate potential directions that require further verification. The question marks denote a process awaiting clarification. (**b**) Created with BioRender.com. Retrieved from https://BioRender.com/z9y8nt9 on 20 May 2026. DKD, diabetic kidney disease; eWAT, epididymal white adipose tissue.

Our findings establish a multi-hit model for *UMOD* deficiency under metabolic and surgical stress (Figure 8a). At baseline, no significant differences in survival, growth, or lipid dysregulation were observed between *UMOD^−/^^−^* and *UMOD^+/+^* rats, indicating that *UMOD* deficiency alone is neither deleterious nor fatal under steady-state conditions. However, under diabetic and surgical stress, *UMOD^−/^^−^* rats showed markedly more severe adipose atrophy and higher mortality. Thus, *UMOD* deficiency does not disrupt basal lipid homeostasis but instead predisposes the organism to multi-hit insults triggered by combined metabolic and surgical stress.

We report that *UMOD* deficiency induces a lipodystrophy-like phenotype characterized by increased UCP1 expression with browning, impaired lipid storage, and eventually fibrosis in diabetes (Figure 8b). WAT browning has context-dependent dual effects [16,17,18]. Under physiological conditions such as exercise or cold exposure, it promotes thermogenesis and energy expenditure, conferring metabolic benefits [16]. However, under pathological conditions such as cancer, severe burns, diabetes, chronic heart failure, and critical illness, WAT browning takes a detrimental turn, leading to lipid droplet loss and excessive energy expenditure, ultimately resulting in weight loss and even death [18]. Multiple studies have demonstrated that circulating molecules play important roles in WAT browning under pathological conditions, including IL-6 [19], parathyroid hormone-related protein [20], TNF-α [21], and natriuretic peptides [22]. Uromodulin is a kidney-specific glycoprotein that is secreted bidirectionally into urine and circulation [5]. Based on this property, we reasoned that uromodulin may exert its metabolic effects on distal adipose tissues via the circulatory route. Future studies are needed to experimentally test this hypothesis.

Our findings help distinguish the mechanism of *UMOD* deficiency from that of *UMOD* gain-of-function mutations [23]. Previous studies reported that mice homozygous for the Umod^A227T^ mutation exhibit renal function decline accompanied by significant lipid abnormalities, with the latter occurring in the context of impaired kidney function [23]. In contrast, *UMOD* deficiency drives lipid dysregulation in the absence of kidney dysfunction. Our findings identify uromodulin as a potential key mediator in a kidney–adipose axis that actively regulates systemic blood lipids. This challenges the traditional view of the kidney as a passive organ and underscores its active role in systemic metabolic regulation. *UMOD* deficiency exacerbates tubular injury and promotes renal lipid accumulation in DKD. Notably, the observation that hyperlipidemia preceded tubular injury (as indicated by uKIM-1) raises the possibility that hyperlipidemia is a candidate risk factor for DKD progression in this model, though causality has yet to be proven.

The finding that uromodulin plays a unique role in adipose tissue homeostasis is surprising and expands our understanding of pathological lipid dysregulation in diabetes. However, several limitations should be acknowledged. First, this study establishes temporal association but not mechanistic causality. Whether the observed adipose atrophy causes subsequent mortality, renal lipid accumulation, and kidney injury remains to be determined. Additionally, the hypothesis that circulating uromodulin acts on eWAT, while plausible given its secretory nature, requires experimental validation. Second, tissue from spontaneously deceased animals was not available for necropsy due to postmortem autolysis. Consequently, the exact causes of death remained undetermined. Third, a multi-hit strategy combining unilateral nephrectomy (UNx), streptozotocin (STZ), and high-fat diet (HFD) was used to induce diabetes and accelerate DKD pathology in this model. It introduced potential confounders that precluded definitive determination of the dominant driver of the observed phenotypes. Fourthly, the sample sizes varied and were relatively small for some histological and biochemical analyses due to random subsampling. Nevertheless, baseline comparability, within-group homogeneity, and random selection support the validity of these findings. Lastly, our findings are derived from animal models, and their translational relevance to human diabetic kidney disease remains to be established. Future work is warranted to fill these gaps.

In conclusion, we show that uromodulin protects against death, eWAT browning, and kidney injury in diabetic rats, establishing it as a novel regulator of the kidney–adipose axis. Further work is needed to uncover the mechanism by which uromodulin acts on adipose tissue homeostasis.

## 4. Materials and Methods

### 4.1. UMOD Knockout Rat Model

*UMOD^−/^^−^* Sprague Dawley (SD) rats were established as previously described [24].

### 4.2. Animal Housing

In this study, specific pathogen-free (SPF) male wild-type (*UMOD^+/+^*) SD rats and *UMOD^−/^^−^* SD rats were used. All animals were bred in-house at the Animal Facility of Peking University First Hospital. Housing conditions were controlled, with constant temperature (25 ± 1 °C), humidity (40–80%), and a 12 h light/dark cycle. All rats were provided free access to a standard maintenance diet (containing 12% fat) and animal-grade purified water.

### 4.3. Rats Under Baseline Conditions

Under baseline conditions, *UMOD^+/+^* and *UMOD^−/^^−^* rats (initial age 6 weeks; *n* = 6 per group) were maintained under the housing and feeding conditions described above and were sacrificed at 24 weeks of age. Body weight, survival, and blood glucose were monitored regularly throughout the study period.

### 4.4. Diabetic Rat Model

Twenty male *UMOD^+/+^* and twenty male *UMOD^−/^^−^* SD rats (6 weeks old) were randomly assigned to either a blank control group (*n* = 6 per genotype) or a DM group (*n* = 14 per genotype). DM rats were fed a HFD (D12492, 60% kcal fat, Research Diets, New Brunswick, NJ, USA) for two weeks, followed by UNx. Two weeks after surgery, they received an intraperitoneal injection of 35 mg/kg STZ (S0130, Sigma, St. Louis, MO, USA). HFD feeding was maintained throughout the experimental period. The blank control group received a standard diet, sham surgery, and vehicle injection in parallel with DN group. The day of STZ injection was defined as day 0, and all rats were sacrificed at week 7. Body weight and food intake were measured regularly throughout the experiment. Animal status and survival were monitored daily, and survival curves were generated. Diabetes modeling was deemed successful upon the fulfillment of the following criteria: (1) manifestation of overt diabetic symptoms, and (2) either fasting blood glucose > 11.1 mmol/L (on two separate occasions) or random blood glucose > 16.7 mmol/L at 72 h after STZ injection. Rats in DM group that did not successfully develop diabetes were excluded from statistical analysis. Any animal showing a body weight loss of >15% relative to its initial weight was euthanized and recorded as deceased for survival analysis. The experiment was terminated for the entire study if the group mortality rate exceeded 35%. All experimental procedures were approved by the Institutional Animal Care and Use Committee of Peking University First Hospital and were conducted in accordance with relevant guidelines for the management and use of laboratory animals.

### 4.5. Fasting Blood Glucose Measurement

Fasting blood glucose was measured after a 12 h fast by collecting a drop of blood from the tail tip using a hand-held glucometer (Accu-Chek Performa, Roche Diagnostics, Mannheim, Germany) according to the manufacturer’s protocol.

### 4.6. Serum and Tissue Lipid Measurements

Blood samples were collected into anticoagulant tubes for plasma separation or into plain tubes for serum separation. For plasma, tubes were centrifuged at 2000× *g* for 15 min at 4 °C; for serum, tubes were allowed to clot for 30 min at room temperature before centrifugation at 3000× *g* for 10 min. Serum levels of TGs and TCHO were measured using commercial kits (TGs: A110-1-1 and TCHO: A111-1-1, Nanjing Jiancheng Bioengineering Institute, Nanjing, China) as it is indicated in the manufacturer’s protocol. Plasma NEFA levels were determined using a corresponding kit (A042-2-1, Nanjing Jiancheng Bioengineering Institute, Nanjing, China). For tissue analysis, kidney and liver samples were homogenized, and TG and TCHO contents were measured using the same kits as described above for serum measurements. Tissue lipid levels were normalized to protein concentration determined by bicinchoninic acid (BCA) assay (WB6501, NCM Biotech, Suzhou, China).

### 4.7. Measurement of Scr, BUN, and ALT

Scr, BUN, and plasma ALT levels were measured using commercial kits (Scr: C011-2-1 and ALT: C009-2-1, Nanjing Jiancheng Bioengineering Institute, Nanjing, China; BUN: E-BC-K329-S, Elabscience, Wuhan, China) according to the manufacturers’ instructions.

### 4.8. Sample Collection and Enzyme-Linked Immunosorbent Assay (ELISA) Measurements

Rats were placed in individual metabolic cages for 12 h to collect urine samples. Water was provided ad libitum, while food was withheld during the collection period. Urine was centrifuged at 3000× *g* for 10 min at 4 °C. Blood samples were collected as described above. All samples were stored at −80 °C until analysis. Following the manufacturers’ instructions, Commercial ELISA kits were used to measure serum insulin (E-EL-R3034, Elabscience, Wuhan, China), adiponectin (E-EL-R3012, Elabscience, Wuhan, China), leptin (SEKR-0051, Solarbio, Beijing, China), IL-6 (E-HSEL-R0004, Elabscience, Wuhan, China), IL-1β (E-HSEL-R0002, Elabscience, Wuhan, China), and TNF-α (E-HSEL-R0001, Elabscience, Wuhan, China), as well as serum and urinary uromodulin (ab274405, Abcam, Cambridge, UK), and urinary KIM-1 (E-EL-R3019, Elabscience, Wuhan, China) and albumin (E111-125, Bethyl Laboratories, Montgomery, TX, USA). Urinary KIM-1, uromodulin, and albumin levels were normalized to urinary creatinine concentration. Urinary creatinine was measured using the same method as described for serum creatinine.

### 4.9. Histology and Morphological Analysis

#### 4.9.1. H&E Staining

Pancreas, liver, and eWAT samples were collected from all rats, fixed in 4% paraformaldehyde, embedded in paraffin, and sectioned into 3-μm thick sections. The sections were stained with H&E (BL700A, Biosharp, Hefei, China) following standard protocols. Whole-slide images were acquired using a slide scanner (SQF-120Pro, Shenzhen, China). The average islet area was calculated from at least 10 randomly selected islets per section using ImageJ software (Version 1.53m, NIH, Bethesda, MD, USA). Adipocyte diameter was measured from at least 100 adipocytes per section, and the adipocytes were categorized into diameter groups (<20 μm, 20–40 μm, 40–80 μm, 80–120 μm, and >120 μm). The percentage of adipocytes in each diameter category was calculated per section, and these values were used for statistical analysis. All scoring was performed in a blinded manner by two independent observers. ImageJ software (Version 1.53m, NIH, Bethesda, MD, USA) was used for the analysis.

#### 4.9.2. PAS Staining

Kidney tissue samples were collected from all rats, fixed in 4% paraformaldehyde, embedded in paraffin, and sectioned into 3-μm thick sections. For PAS staining (BA4114, Baso, Zhuhai, China), the sections were deparaffinized, rehydrated, and oxidized with 0.5% periodic acid for 10 min. After washing, the sections were incubated with Schiff’s reagent for 20 min at room temperature in the dark, and counterstained with hematoxylin. The sections were then dehydrated, cleared, and mounted. Whole-slide images were acquired using a slide scanner (SQF-120Pro, Shenzhen, China). For each kidney section, at least 20 randomly selected glomeruli were assessed for mesangial expansion using a semi-quantitative scoring system: 0, normal (no expansion); 1, mild expansion (<25% of the glomerular tuft); 2, moderate expansion (25–50%); 3, severe expansion (>50%); and 4, global sclerosis. The mesangial expansion index for each rat was calculated as the average score across all glomeruli examined. Tubular injury was evaluated in 10 randomly selected non-overlapping fields per section at 200× magnification. Injured tubules, defined as those showing tubular dilation, atrophy, cast formation, or epithelial detachment, were traced. The injured area relative to the total field area was calculated as the tubular injury index: (injured tubular area/total field area) × 100%. The average index of the 10 fields was used for statistical analysis. All scoring was performed in a blinded manner by two independent observers. ImageJ software (Version 1.53m, NIH, Bethesda, MD, USA) was used for the analysis.

#### 4.9.3. Masson’s Trichrome Staining

Adipose tissue sections were prepared as described above. Masson’s trichrome staining (G1340, Solarbio, Beijing, China) was performed on adipose tissue sections following standard protocols. Briefly, sections were rehydrated, stained with Weigert’s iron hematoxylin, followed by Biebrich scarlet-acid fuchsin, differentiated in phosphomolybdic-phosphotungstic acid, and finally stained with aniline blue. After dehydration and clearing, sections were mounted and visualized using a light microscope (Olympus, Tokyo, Japan). Whole-slide images were acquired using a slide scanner (SQF-120Pro, Shenzhen, China). The fibrotic area was quantified using ImageJ software (Version 1.53m, NIH, Bethesda, MD, USA) by calculating the percentage of blue-stained area relative to the total tissue area. All scoring was performed in a blinded manner by two independent observers.

#### 4.9.4. Sirius Red Staining

Kidney tissue sections were prepared as described above. Sirius Red staining (G1472, Solarbio, Beijing, China) was performed on kidney sections to evaluate collagen deposition and interstitial fibrosis. According to the manufacturer’s instructions, sections were incubated in 0.1% Picro-Sirius Red solution for 60 min. After rinsing in 0.5% acetic acid solution and dehydration through graded alcohols, sections were cleared and mounted. For quantitative analysis, images were acquired using a slide scanner (SQF-120Pro, Shenzhen, China). The red-stained collagen fibers under brightfield and the birefringent fibers under polarized light were measured as the percentage of fibrotic area using ImageJ software (Version 1.53m, NIH, Bethesda, MD, USA). All morphometric analyses were performed in a blinded fashion.

### 4.10. Immunofluorescence Staining

Paraffin-embedded kidney and adipose tissue sections were deparaffinized in xylene and rehydrated through a graded ethanol series. For antigen retrieval, sections were immersed in sodium citrate buffer (10 mM, pH 6.0) and heated in a pressure cooker for 3 min. After cooling to room temperature, sections were washed with PBS, permeated with 0.5% Triton X-100 in PBS for 10 min at room temperature, and then blocked with 3% bovine serum albumin (BSA) in PBS for 1 h at 37 °C. Sections were then incubated overnight at 4 °C with primary antibodies: anti-uromodulin (ab207170, Abcam, Cambridge, UK) for kidney sections, and anti-UCP1 (ab209483, Abcam, Cambridge, UK) for adipose tissue sections. After washing with PBS, sections were incubated with fluorophore-conjugated secondary antibodies (A0516, Beyotime, Shanghai, China) for 1 h at 37 °C in the dark. Nuclei were counterstained with DAPI (ab104139, Abcam, Cambridge, UK). Images were acquired using a slide scanner (SQF-120Pro, Shenzhen, China). UCP1-positive areas were quantified as the percentage of positive area per total tissue area using ImageJ software (Version 1.53m, NIH, Bethesda, MD, USA). All scoring was performed in a blinded manner by two independent observers.

### 4.11. Immunohistochemistry

Paraffin-embedded kidney sections were deparaffinized in xylene and rehydrated through a graded ethanol series. For antigen retrieval, sections were immersed in sodium citrate buffer (10 mM, pH 6.0) and heated in a pressure cooker for 3 min. After cooling to room temperature, sections were washed with PBS. Endogenous peroxidase activity was quenched with 3% H_2_O_2_ in PBS for 15 min at room temperature. Sections were then blocked with 5% BSA in PBS for 1 h at 37 °C and incubated overnight at 4 °C with primary antibodies: anti-α-SMA (BM0002, Boster, Wuhan, China). After washing with PBS, sections were incubated with HRP-conjugated secondary antibody for 1 h at 37 °C. Staining was developed using DAB substrate. Sections were counterstained with hematoxylin, dehydrated, cleared, and mounted. Images were acquired using a slide scanner (SQF-120Pro, Shenzhen, China). The positive areas were quantified as a percentage of the total tissue area using ImageJ software (Version 1.53m, NIH, Bethesda, MD, USA). All scoring was performed in a blinded manner by two independent observers.

### 4.12. Quantitative Real-Time PCR (qPCR) Analyses

Tissue samples were homogenized in TRIzol reagent (15596026, Thermo Fisher Scientific, Waltham, MA, USA) for total RNA extraction. Complementary DNA was synthesized from 1 μg of total RNA using the HiScript III All-in-one RT Supermix (R333, Vazyme, Nanjing, China). The primer pairs (Table 1) were verified to have reliable amplification efficiency. PowerUp SYBR Green Master Mix (A25742, Thermo Fisher Scientific, Waltham, MA, USA) was employed for qPCR on an Applied Biosystems 7500 Fast Real-Time PCR System (Thermo Fisher Scientific, Waltham, MA, USA). β-actin was used as the reference gene, and target gene expression was normalized using the 2^−△△Ct^ method.

### 4.13. Bioinformatics Analysis

To investigate the transcriptional changes in the *UMOD* gene in DKD, public databases were analyzed. The GSE30529 dataset was obtained from Gene Expression Omnibus (GEO) as a pre-processed series matrix file [25,26]. The expression matrix contained 22 human kidney samples derived from tubulointerstitium and vascular compartments (10 DKD patients and 12 healthy controls). The data had been previously normalized by the submitter using the Robust Multi-array Average (RMA) algorithm. Therefore, no additional normalization was applied. Probes without corresponding gene symbols were removed, and for genes with multiple probes, the maximum expression value was used. Differential expression analysis was performed using the limma package with the Benjamini-Hochberg method for false discovery rate (FDR) control. Differentially expressed genes (DEGs) between DKD and control samples were defined as those with |log_2_ fold-change| > 0.585 (>1.5-fold) and FDR < 0.05. To investigate uromodulin function, samples were stratified into high- and low-*UMOD* expression groups by the median *UMOD* level. Differential expression between these two groups was performed using the limma package, and all genes were ranked by log_2_ fold-change (without filtering) as input for pre-ranked gene set enrichment analysis (GSEA) using the clusterProfiler package and Kyoto Encyclopedia of Genes and Genomes (KEGG) gene sets. Statistical significance for GSEA was defined as a Benjamini-Hochberg-adjusted *p* < 0.05. All bioinformatics analyses were performed using RStudio (version 4.2.1).

### 4.14. Statistical Analysis

Statistical analysis was performed with GraphPad Prism (Version 8.0.2, GraphPad Software, LLC, Boston, MA, USA). Normality was tested for all data sets using the Shapiro–Wilk test. For two-group comparisons, data following a normal distribution are presented as mean ± SD and were analyzed using Student’s *t*-test, while non-normally distributed variables are expressed as median with interquartile range and were compared using the Mann–Whitney U test. For multi-group comparisons, one-way ANOVA with Tukey’s multiple comparisons test was applied. Survival curves were plotted using the Kaplan–Meier method and compared using the log-rank test. Pearson correlation analysis was used to assess the correlation between sUMOD and uUMOD/Cr. Available case analysis was used for longitudinal outcomes. Missing data due to death were not imputed. A two-tailed *p*-value of less than 0.05 was considered statistically significant.

## Figures and Tables

**Table 1:** Primer sequences used for qPCR.

Gene	Forward Primer	Reverse Primer
*ACTB*	CCTAGACTTCGAGCAAGAGA	GGAAGGAAGGCTGGAAGA
*UMOD*	CTGGACATGAAAGTCAGTCTGAAGA	CCACCCAAGCTGATGTTCAA

## Data Availability

The original contributions presented in this study are included in this article. Further inquiries can be directed to the corresponding author.
